# Inhibition of Wilms' Tumor Proliferation and Invasion by Blocking TGF-*β* Receptor I in the TGF-*β*/Smad Signaling Pathway

**DOI:** 10.1155/2020/8039840

**Published:** 2020-11-16

**Authors:** Qinlin Shi, Huan Wu, Yonglin Li, Lianju Shen, Xiaomao Tian, Tao Lin, Guanghui Wei

**Affiliations:** ^1^Ministry of Education Key Laboratory of Child Development and Disorders, Chongqing Key Laboratory of Pediatrics, Chongqing Key Laboratory of Children Urogenital Development and Tissue Engineering, China International Science and Technology Cooperation Base of Child Development and Critical Disorders, Pediatric Research Institute, Children's Hospital of Chongqing Medical University, Chongqing 400014, China; ^2^Department of Pediatric Urology Surgery, Children's Hospital of Chongqing Medical University, Chongqing 400014, China

## Abstract

Wilms' tumor (WT) is a common embryonal tumor, and nephrogenic rests play a critical role in WT development. The transforming growth factor *β* (TGF-*β*) signaling pathway is fundamental to embryo development and cell growth and proliferation. Moreover, TGF-*β* contributes to WT development, but the mechanisms of disease pathogenicity are unknown. This study investigated whether the TGF-*β* signaling pathway was involved in WT and whether blocking T*β*RI receptor inhibited WT growth, proliferation, and invasion. A total of 60 WT patients with clinical data and surgical specimens were evaluated. Immunohistochemistry (IHC) was used to detect the expression of TGF-*β*1 and P-smad2/3. In vitro, the proliferation, migration, apoptosis, and epithelial-mesenchymal transition (EMT) protein expression were analyzed using the CCK8 assay, wound healing assay, transwell assay, flow cytometry, and western blot, respectively. In vivo, tumor morphology, tumor size, toxicity, and EMT protein expression were analyzed in tumor-bearing mice treated with a T*β*RI kinase inhibitor or PBS. High protein levels of TGF-*β*1 and P-samd2/3 were associated with clinical stage and metastasis or invasion. T*β*RI inhibition effectively suppressed WT proliferation and migration and promoted apoptosis in the human WT cell line G401, consequently decreasing EMT protein expression. In addition, the T*β*RI kinase inhibitor significantly impaired the subcutaneous growth of WT. It is worth noting that treatment with the T*β*RI kinase inhibitor did not cause liver and kidney injury. Our results indicate that the TGF-*β*/Smad signaling pathway plays a crucial role in WT progression. Blocking the T*β*RI receptor may be a novel strategy to treat and prevent WT.

## 1. Introduction

Wilms' tumor (WT) is a common malignant embryonal tumor of the kidneys, accounting for 6% of all malignant tumors in children [1]. The overall survival rate in WT is approximately 90% using multidisciplinary treatments (surgery, radiation, and chemotherapy) and individualized treatment [2]. However, prognosis is poor in some cases because of metastasis, recurrence, and anaplastic WT [3–5]. Moreover, treatment causes severe long-term side effects, including musculoskeletal complications, cardiac toxicity, renal dysfunction, reproductive problems, and second malignant neoplasms [6].

Although many studies suggest that the pathogenesis of WT may involve changes in multiple genes (WT1, WTX, MYCN, IGF2, CTNNBA, SIX1/SIX2, and TP53) [7], loss of heterozygosity, and familial genetic factors (Denys-Drash syndrome, Beckwith-Wiedemann syndrome, and Perlman syndrome) [8], the mechanisms underlying pathogenicity are unclear.

The transforming growth factor beta (TGF-*β*) signaling pathway controls complex biological functions which involve in organ development, cell proliferation and differentiation, and immune modulation [9, 10]. TGF-*β* signal transduction is mediated by a pair of transmembrane serine/threonine kinase receptors. Activated TGF-*β* binds to TGF-*β* receptor II (T*β*RII) homodimers, allowing the attachment of two T*β*RI molecules to heterotetrameric complexes, and resulting in the activation of T*β*RI kinase by T*β*RII kinase [11]. Furthermore, Smad2 and Smad3 transiently bind to and are phosphorylated by T*β*RI kinase, allowing these Smad proteins to form heteromeric complexes with Smad4 [12]. Studies demonstrated that the dysregulation of the TGF-*β* signaling pathway might result in tumor development, and suppression of T*β*RI can inhibit tumor growth [13, 14]. In pediatric tumors, particularly WT, abnormally activated TGF-*β* signaling may be an important mediator of tumor cell growth, and the positive expression of TGF-*β* in WT was associated with tumor anaplastic, invasion, and disease progression [15–17]. Although TGF-*β* signaling played a key role in WT, the molecular mechanisms behind this regulation are unknown [18].

This study determined the expression profile of TGF-*β*/Smad in WT and adjacent normal tissues to elucidate the mechanism WT invasion and metastasis. To investigate the mechanism WT development, the TGF-*β*/Smad signaling pathway and EMT were inhibited by blocking T*β*RI. It is worth stressing that T*β*RI inhibition may constitute a novel strategy for treating WT.

## 2. Materials and Methods

### 2.1. Tissue Samples

A total of 60 pairs of WT tissues and adjacent normal tissues were harvested from patients who underwent partial or radical nephrectomy at the Department of Urology of Children's Hospital of Chongqing Medical University. The diagnosis was based on pathological evidence, and the collected tissue samples were immediately snap-frozen in liquid nitrogen until use. The study was approved by the Research Ethics Committee of our hospital, and all study participants signed an informed consent form. The clinicopathological characteristics of these patients are summarized in Table 1.

### 2.2. Immunohistochemistry

Immunohistochemistry (IHC) was used to detect the expression of TGF-*β*1 and P-Smad2/3. IHC was performed using anti-TGF-*β*1 monoclonal antibody (Abcam, clone number ab92486) and anti-P-smad2/3 monoclonal antibody (1 : 200, Santa Cruz Biotechnology, clone number sc-11769-R). Membranous TGF-*β*1 and nuclear P-smad2/3 expression in tumor cells were quantified using an *H*-score, according to the previous described [19], which takes into consideration the percentage of positive tumor cells within each staining category (0 = negative, 1 = weak, 2 = moderate, 3 = strong). Positivity was defined as the positive signal detected on tumor cells with stainingintensity ≥ 1. Two experienced genitourinary pathologists independently assessed all IHC images.

### 2.3. Cell Cultures

The human WT cell line G401 was purchased from the Beijing Union Cell Resource Center. G401 cells were cultivated in McCoy's 5A (Gibco, USA) medium supplemented with 15% fetal bovine serum (FBS) (modified, Gibco, USA), 100 IU/ml penicillin, and 100 *μ*g/ml streptomycin in a 5% CO_2_ incubator at 37°C.

### 2.4. Cell Proliferation Assays

Cells were plated at 1 × 10^4^ per well in 96-well cluster dishes (Corning, Inc., Corning, NY). After 24 h, the cultures were treated with different doses (0, 1, 2, 5, and 10 *μ*mol/ml) of a T*β*RI kinase inhibitor (MCE, HY-13227), a TGF-*β* agonist (0, 1, 2, 5, and 10 *μ*l) (MCE, SRI-011381), and dimethyl sulfoxide (DMSO) (0, 1, 2, 5, and 10 *μ*l). After 24 hours of incubation, CCK8 was added to the plate wells and incubated at 37°C for 4 h. Absorbance was read in a microplate reader at an optical density of 450 nm.

### 2.5. Cell Apoptosis Assays

G401 cells in the logarithm stage (1 × 10^5^) were cultured in 6-well plate. When the cells reached 80%, the treatment was performed with 2 *μ*mol/ml T*β*RI kinase inhibitor, 2 *μ*mol/ml TGF-*β* agonist, and 2 *μ*mol/ml DMSO, respectively. Sufficient complete medium was added for 24 h. Single cell suspension was prepared and centrifuged at 1000 rpm for 5 min. The supernatant was discarded, and cells were resuspended with PBS, and then, cell counting was performed. In addition, 5 × 10^5^ cell suspension was centrifuged at 1000 rpm for 5 min, and then, the supernatant was removed. Cells were washed in 4°C PBS and incubated with Annexin V-FITC/PI (5 *μ*l, Boster, Wuhan, MK1028) for 15 min in the dark, and cell apoptosis was analyzed by flow cytometry within 1 h. The assay was performed three times independently.

### 2.6. Cell Invasion Assay

Invasion assays were performed using Matrigel-coated Transwell chambers (Corning, New York, NY, USA). Migration assays were performed using Transwell chambers without the Matrigel coating. A total of 1 × 10^4^ cells were harvested in serum-free medium and placed in the upper chamber, and the medium containing 15% FBS was placed in the lower chamber and served as a chemoattractant. Cell cultures were treated as described above. After 24 h of incubation, invasive cells were fixed with methanol and stained with 0.1% crystal violet and photographed under a microscope. Cells were counted using a Beckman Coulter counter.

### 2.7. Wound Healing Assay

Cells were seeded at a density of 1 × 10^6^ cells/well for 24 h. The confluent monolayer of cells was wounded using a 10 *μ*l pipette tip. The cell cultures were treated with 2 *μ*mol/ml T*β*RI kinase inhibitor, 2 *μ*mol/ml TGF-*β* agonist, and 2 *μ*mol/ml DMSO. The existing culture medium was replaced with fresh McCoy's 5A medium supplemented with 15% FBS, streptomycin (100 mg/ml), and penicillin (100 IU/ml). Three images were captured per well using a microscope at 4 and 24 h after wounding. Images were taken of nonoverlapping fields in each well at 0, 4, and 24 after the scratching step using the ImageJ software.

### 2.8. Western Blotting

Proteins were extracted from cultured cells, and tumor tissues of tumor-bearing mice using, respectively, 0.1 ml and 1.0 ml radioimmunoprecipitation assay reagent containing 1% phenylmethanesulfonyl fluoride to prevent protein degradation. Protein extracts were subjected to SDS-PAGE and analyzed using the following primary antibodies: TGF-*β*1, Smad2/3 (sc-398844, Santa Cruz Biotechnologies), P-smad2/3, E-cadherin (ab1416, Abcam), N-cadherin (ab6528, Abcam), cytokeratin (CK) (ab9377, Abcam), *β*-catenin (ab32572, Abcam), *α*-SMA (ab124964, Abcam), MMP-7 (ab5706, Abcam), and *β*-actin (TA-09; ZSGB-BIO, Beijing China). After that, the membranes were incubated with secondary antibodies at 4°C overnight, washed in PBST thrice for 10 min each time, and incubated with the corresponding secondary antibody. The dilution ratio was determined as recommended. Protein levels were measured using the Image-Pro Plus system version 6.0. Band intensity was quantified using the ImageJ software version 1.34. All experiments were performed in triplicate.

### 2.9. Animals

Nude mice aged 4–6 weeks were purchased from the Chongqing Medical University Lab Animal Research Center (Chongqing, China). All animal procedures were approved by the Animal Care and Use Committee of Chongqing Medical University and were performed strictly in accordance with institutional policies. The animals were weighed and randomly assigned to different treatment groups.

A total of 1 × 10^7^ tumor cells were harvested in 0.2 ml phosphate-buffered saline (PBS) and injected subcutaneously into the right upper back of mice. After that, mice were treated with a single daily dose (0.1 ml) of vehicle (1% (*w*/*v*) methylcellulose SD-208 (60 mg/kg)) or 0.1 ml PBS by gavage beginning at day 1 after cell inoculation. The tumor volume in each mouse was measured every three days using an external caliper. Eight weeks after injection, mice were anesthetized and subcutaneously injected with 75 mg/kg D-luciferin (Xenogen, USA) in PBS.

Blood samples were obtained from each mouse; measuring index includes alanine aminotransferase (ALT), aspartate aminotransferase (AST), serum creatinine (Cr), and blood serum uric acid nitrogen (BUN). The serum ALT/AST, Cr, and BUN were analyzed using a blood biochemistry analyzer (Department of Clinical Laboratory, Children Hospital of Chongqing Medical University).

Tumor-bearing mice were sacrificed by CO_2_ asphyxiation, and the tumors were dissected. Tumor volume (*V*) was calculated as *V* = (length × width^2^ × 0.52).

### 2.10. Hematoxylin-Eosin Staining and Immunofluorescence

The tumor tissues were routinely fixed in 4% paraformaldehyde and embedded in paraffin. Subsequently, the 4 *μ*m tumor sections were dewaxed using xylene and rehydrated in graded alcohols. Mounted slides were dipped into hematoxylin, agitated for 2 min, and then rinsed in distilled water for 1 min. The slides were subsequently stained with 1% eosin solution for 3 s with agitation. The sections were dehydrated twice in 95% alcohol (3 min each time) and then twice in 100% alcohol (3 min each time). Finally, the alcohol was extracted with two changes of xylene. Images were acquired on an Olympus microscope.

Frozen tumor tissues were sectioned into 10 *μ*m sections using a cryostat microtome. The samples were fixed in 4% paraformaldehyde for 15 min and permeabilized in 0.1% Triton X-100 in PBS for 15 min. The sections were blocked in 0.5% bovine serum albumin in PBS for 1 h at room temperature and incubated overnight with the primary antibodies N-cadherin, E-cadherin, pan-CK, and *β*-catenin at 4°C. The sections were washed in PBS and incubated with goat anti-rabbit Cy3-conjugated antibody and anti-mouse FITC-conjugated antibody (CwBio, China) for 1 h at room temperature. The nuclei were stained with Hoechst 33342 (C1022, Beyotime, China) for 1 h. Images were obtained using an A1R confocal microscopy system (Nikon, Tokyo, Japan) and analyzed in the NIS-Element AR software version 4.0 (Nikon, Tokyo, Japan).

### 2.11. Statistical Analysis

Each experiment was repeated at least three times. Statistical analyses were performed using SPSS version 17.0 (Chicago, USA). Data were expressed as means ± standarddeviation. Student's *t*-test was used to evaluate the differences between two groups; one-way analysis of variance (ANOVA) was used to assess differences among multiple groups, and the Spearman test was used for correlation analysis. *P* values smaller than 0.05 were considered statistically significant.

## 3. Results

### 3.1. Clinicopathological Findings

From December 2015 to December 2019, 60 patients (35 men and 25 women, with a mean age of 34.3 months) from the Children Hospital of Chongqing Medical University were included in the study. Of these, 47 patients had favorable histology, and 13 patients had unfavorable histology. The number of patients with stages I to IV was 12, 10, 28, and 10, respectively. The invasion and metastasis group of 24 patients was defined as local invasion and metastasis.

### 3.2. High Expression of TGF-*β*1 and P-Smad2/3 in WT Was Associated with Advanced Staging and an Invasive/Metastatic Phenotype

**Figure 1:**
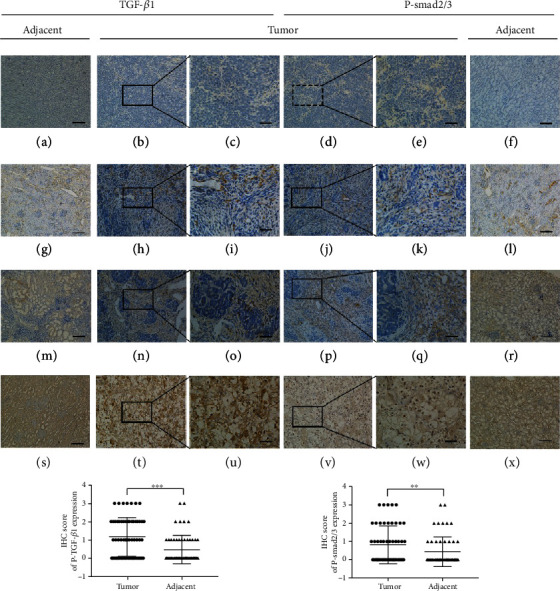
High expression of TGF-*β*1 and P-Smad2/3 in Wilms' tumor tissue. Immunohistochemical staining for TGF-*β*1 and P-Smad2/3 in Wilms' tumors and adjacent normal tissues. (a–f), (g–l), (m–r), and (s–x) represent the specimen from the same patient, respectively. (a–f) Negative expression of TGF-*β*1 and P-Smad2/3 proteins. (g–l, m–k, and s–x) Staining intensity (weak, moderate, strong) for TGF-*β*1 and P-Smad2/3. Compared with the adjacent normal tissues, the expression of TGF-*β*1 and P-Smad2/3 was significantly increased (^∗∗∗^*P* < 0.001, ^∗∗^*P* < 0.01).

To determine the potential predictive role of TGF-*β*/Smad in WT, the expression of TGF-*β*1 and P-Smad2/3 was measured in 60 paired WT and adjacent normal tissues by IHC staining (Figure 1). Compared with adjacent tissues, the TGF-*β*1 and P-Smad2/3 expression was significantly upregulated in 38 (63.3%) and 26 (43.3%) WT specimens, respectively (Figure 1). Furthermore, the expression of TGF-*β*1 and P-Smad2/3 in stages III and IV was significantly higher than that in stages I and II (Table 1). The percentage of samples positive for TGF-*β*1 and P-Smad2/3 was 79.2% (19/24) and 66.7% (16/24), respectively, in the group with an invasive/metastatic phenotype and 52.7% (19/36) and 27.8% (10/36), respectively, in the group without this phenotype (Table 1).

Moreover, the P-smad2/3 expression was positive correlated with TGF-*β*1 in WT tissues (*R* = 0.617, *P* < 0.001). Collectively, these results demonstrate that the TGF-*β*1 and P-smad2/3 expression is upregulated in malignant WT specimens and correlates with advanced tumor staging and invasion/metastasis.

### 3.3. Effects of the T*β*RI Kinase Inhibitor on WT Cell Growth, Apoptosis, Migration, and Invasion

**Figure 2:**
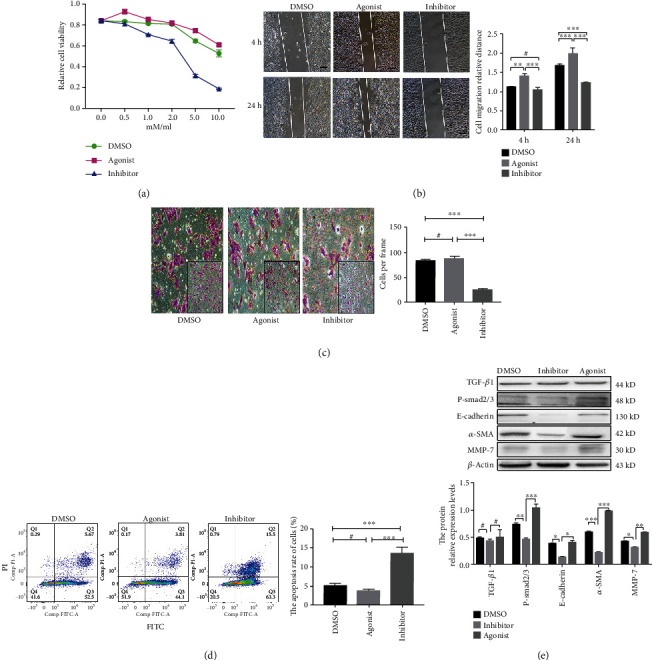
TGF-*β* receptor I (T*β*RI) inhibition decreased epithelial-mesenchymal transition regulated by the TGF-*β*/Smad signaling pathway, resulting in Wilms' tumor proliferation, migration, invasion, and apoptosis. (a) Cell counting kit-8 assay was performed to determine cell proliferation of G401 cells that were treated with different concentrations of T*β*RI inhibitor, TGF-*β* agonist, or DMSO for 4 h. (b) Wound healing assay was performed to determine the migration rate of G401 cells at 4 h and 24 h after treatment with DMSO, T*β*RI inhibitor, or TGF-*β* agonist (magnification, 20x). (c) Transwell-mediated invasion assay was performed to determine the invasion ability of G401 cells which were treated by DMSO, T*β*RI inhibitor, or TGF-*β* agonist. (d) Flow cytometry analysis was performed to determine the apoptosis rate of G401 cells. (e) Western blot was used to determine the expression of TGF-*β*1, P-smad2/3, E-cadherin, *α*-SMA, and MMP-7. GAPDH was used as a loading control. The relative expression was analyzed. (#*P* > 0.05, ^∗^*P* < 0.05, ^∗∗^*P* < 0.01, ^∗∗∗^*P* < 0.001).

To explore the function of inhibition of TGF-*β*/Smad signaling in WT, we investigated the effects of T*β*RI kinase inhibitor targeted to TGF-*β*/Smad on cell proliferation, tumorigenesis, and invasion in G401 cell line. The growth of G401 cells in vitro was significantly impaired by the T*β*RI kinase inhibitor in a dose-dependent manner compared with DMSO (*P* < 0.05, Figure 2(a)). In contrast, treatment with TGF-*β* agonist did not significantly affect cell growth (*P* > 0.05). Considering the cell toxicity effect of DMSO in G401, we chose 2 *u*l/ml concentration for subsequent experiments. Inhibition of T*β*RI significantly inhibited cell growth in G401 cell. Next, a wound healing assay was detected the cell migration. Compared with the DSMO group, the inhibition of T*β*RI dramatically prevented the migration of G401 in 24 hours (*P* < 0.001, Figure 2(b)). In addition to cell invasion, a Matrigel-mediated invasion assay was performed to determine the effects of T*β*RI kinase inhibitor on cell invasion. We found the inhibition of T*β*RI could effectively decreased in cell invasion (*P* < 0.001, Figure 2(c)). Meanwhile, by apoptosis assay, we found T*β*RI kinase inhibitor could cause the apoptosis of G401 cell in the late stage (*P* < 0.001, Figure 2(d)). Taken together, inhibition of T*β*RI plays a critical role in regulating cell proliferation, tumorigenesis, and metastasis in WT cells.

### 3.4. T*β*RI Kinase Inhibitor Suppresses WT Growth In Vivo

**Figure 3:**
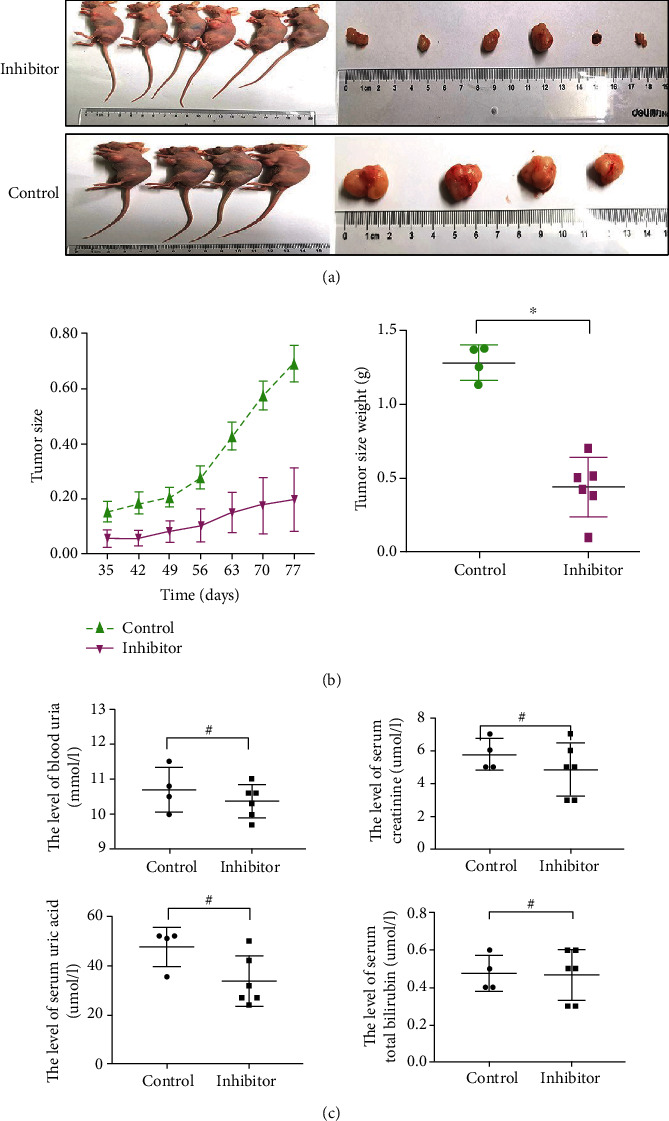
TGF-*β* receptor I (T*β*RI) inhibitor suppressed WT growth in vivo. (a) Representative macroscopic findings of Wilms' tumor. Two tumor-bearing mice from the control group died at day 65 after treatment. Tumor size (^∗∗^*P* < 0.01) and final tumor weight (^∗^*P* < 0.05) of G401 tumor cells after treatment with T*β*RI kinase inhibitor (60 mg/day) or PBS (control). (b) The toxicity of T*β*RI kinase inhibitor was determined by assessing the liver and kidney markers (blood urea nitrogen, serum creatinine, serum uric acid, and total bilirubin). The concentration of these markers was not significantly different between the two groups (#*P* > 0.05).

To establish whether T*β*RI inhibition is a potential therapeutic target, a subcutaneous tumor model was established by injecting G401 cells into the flank of wild-type BALB/c nude mice. Then, mice were treated with a T*β*RI inhibitor by gavage. Tumors treated with the inhibitor grew more slowly and were smaller than control tumors (Figure 3(a)). Meanwhile, two mice in the control group died at day 68 after treatment. Compared with the control group, T*β*RI inhibitor significantly reduced tumor volume and weight (*P* < 0.05, Figure 3(a)). The effect of T*β*R inhibitors on mouse growth was investigated during the period of present study. Compare with the control group, there are no signs of toxicity by the detection of the serum ALT/AST, BUN, and Cr (*P* > 0.05, Figure 3(b)). It means animal samples did not find chemical acute toxicity effect. Collectively, in vivo, the data suggest that T*β*RI kinase inhibitor is a potential therapy target for WT.

### 3.5. T*β*RI Inhibition Reduces EMT by Inhibiting the TGF-*β*/Smad Signaling Pathway

**Figure 4:**
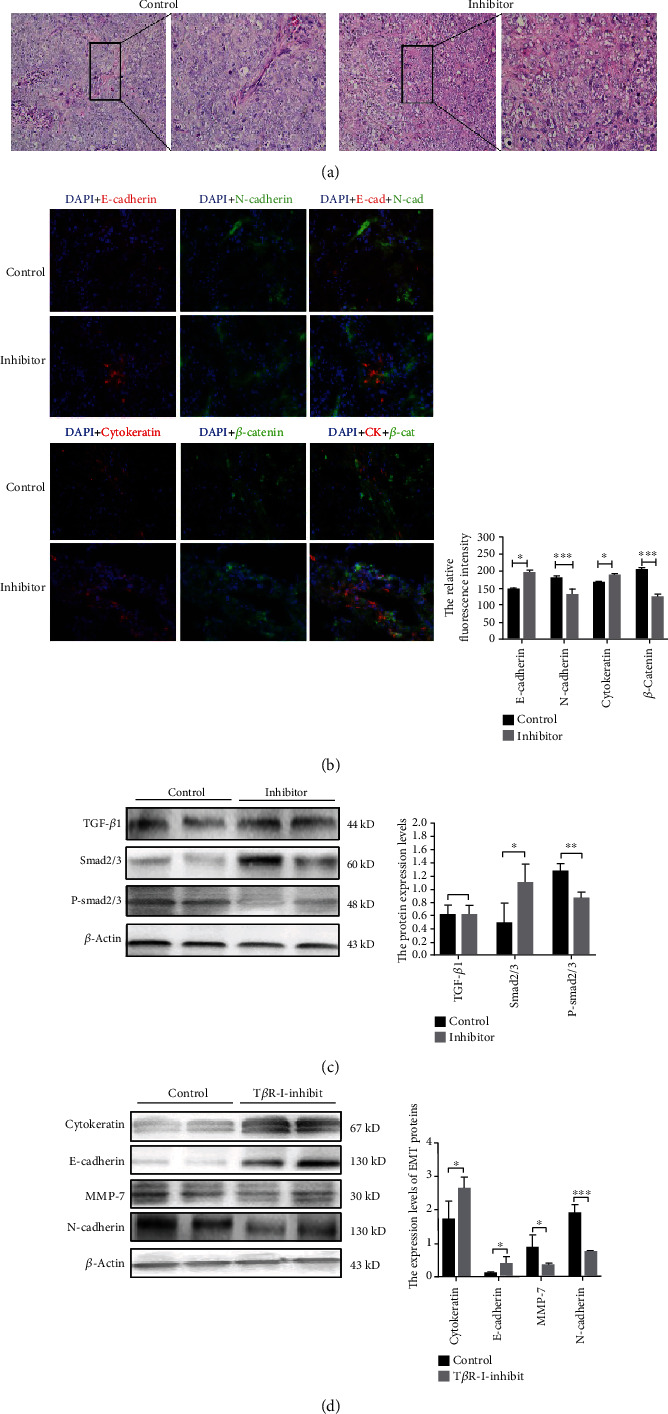
TGF-*β* receptor I (T*β*RI) inhibition reduces EMT in WT in vivo. (a) Hematoxylin-eosin staining in tumor treated with either T*β*RI kinase inhibitor or PBS (control) (magnification, 20 and 40). (b) Double-immunofluorescence showing that T*β*RI inhibition significantly decreased the expression of N-cadherin and *β*-catenin and considerably increased the expression of E-cadherin and cytokeratin (magnification, 40). (c) Western blot showing that the T*β*RI inhibitor significantly upregulate the expression of P-Smad2/3 and decreased the expression of Smad2/3. (d) Western blot of EMT relatively protein expression showed that treatment with the T*β*RI inhibitor significantly upregulated cytokeratin and E-cadherin and downregulated N-cadherin and MMP-7 (^∗^*P* < 0.05, ^∗∗∗^*P* < 0.001).

EMT is a key factor in tumor cell invasion and migration, and TGF-*β*/Smad signaling pathway activation is crucial to tumor EMT. The H&E stain was used to observe the morphological change between T*β*RI inhibitor group and control group. Compared with the control group, cell vacuolation is increased, and the stroma structure loose (Figure 4(a)). Double immunofluorescence and western blotting were used to investigate whether inhibition of T*β*RI could effect the epithelial cells transformed into mesenchymal cells in WT. The western bolt results showed that the protein expression levels of P-Smad2/3, E-cadherin, *α*-SMA, and MMP-7 in the T*β*RI kinase inhibitor group were significantly lower than those in the DMSO group and TGF-*β* agonist group (*P* < 0.05), whereas the TGF-*β*1 expression was not significantly different between these groups (*P* > 0.05, Figure 2(e)). Moreover, in vitro, compared with the control group, the protein expression levels of P-Smad2/3, N-cadherin, and MMP-7 were significantly decreased in the inhibitor group (*P* < 0.05), whereas the protein expression of Smad2/3, CK, and E-cadherin was increased (*P* > 0.05, Figures 4(c) and 4(d)). Similarly, double immunofluorescence results showed the concomitant expression of epithelial markers (E-cadherin and CK) and mesenchymal markers (N-cadherin and *β*-catenin) (Figure 4(b)). Compared with the control group, the expression levels of N-cadherin and *β*-catenin were significantly decreased (*P* < 0.05), and the expression of E-cadherin and CK was upregulated after treated with the T*β*R inhibitor (*P* < 0.05, Figure 4(b)). Taken together, these results demonstrated that T*β*RI inhibition can suppress EMT by inhibiting the TGF-*β*/Smad signaling pathway.

## 4. Discussion

WT is the most common malignant retroperitoneal tumor, accounting for approximately 95% of renal tumors in children. The good prognosis of WT is currently attributed to the continuous efforts of the National Wilms Tumor Study Group and the International Society of Pediatric Oncology [20–22]. However, metastasis, relapse, and chemotherapy resistance also severely influence long-term patient survival [23, 24]. Therefore, it is crucial to explore the mechanisms underlying WT progression, and targeting this mechanism may constitute a promising therapeutical strategy. Several studies have shown that TGF-*β* signaling helps regulate cell development, growth, differentiation, and apoptosis [25], but developing tumors are more responsive to TGF-*β*, which stimulates cell motility, invasion, metastasis, and tumor stem cell maintenance [13, 15].

The dysregulation of TGF-*β* has been linked to the initiation and progression of multiple human cancers, including WT [18]. Several studies have demonstrated that the TGF-*β* signaling was activated by TGF-*β*; the ligands lead to the phosphorylation of Smad proteins [16]. The present study compared the expression of TGF-*β*1 and P-Smad2/3 in WT and adjacent normal tissues. The correlations between clinicopathological parameters and the expression levels of TGF-*β*1 and P-Smad2/3 were also evaluated. The results indicated that the TGF-*β*1 and P-Smad2/3 expression was upregulated in WT. In WT patients, the expression was positively associated with clinical stage and metastasis/invasion but negatively associated with age, gender, and pathology. Furthermore, the expression level of TGF-*β*1 was positively correlated with P-Smad2/3 expression level.

The activation of the TGF-*β*/Smad signaling pathway requires the binding of TGF-*β* to T*β*RII, which then recruits and phosphorylates T*β*RI. T*β*RI phosphorylates SMAD2 and SMAD3, which translocate to the nucleus and regulate cell function. Therefore, T*β*RI and II play a crucial role in the activation of the TGF-*β*/Smad signaling pathway. A recent study proved that T*β*RI kinase inhibitors inhibited the metastatic efficiency and growth of mouse mammary carcinomas [26]. Another study found that systemic treatment with T*β*RI kinase inhibitors could be combined with local approaches to regulate the levels of TGF-*β* in human and mouse glioma [14]. The present study investigated the mechanism of T*β*RI inhibition in vitro and found that T*β*RI kinase inhibitors decreased the proliferation of G401 cells in a dose-dependent manner. T*β*RI inhibition considerably decreased WT migration and invasion and increased apoptosis in G401 cells. Furthermore, treatment with 60 mg/kg/d strongly decreased WT growth in tumor-bearing mice and did not significantly cause kidney and liver injury in mice. Compared with the TGF-*β* agonist and DMSO groups, T*β*RI inhibition reduced the expression of P-smad2/3 but did not change TGF-*β*1 levels.

It is worth noting that TGF-*β* induces EMT in embryonic epithelial cells, and EMT is thought to contribute to tumor invasion and metastasis [27, 28] and is a marker of tumor progression [29]. Epithelial tumor cells acquire an invasive phenotype, characterized by the increased expression of mesenchymal markers such as vimentin, increased secretion of metalloproteinases, and the reduced expression of cell-cell adhesion molecules such as E-cadherin, leading to metastasis [30]. In this study, we demonstrated that T*β*RI inhibition decreased the expression levels of vimentin, N-cadherin, and MMP-7 and increased the levels of E-cadherin and CK both in vivo and in vitro.

Our study has some limitations including; the study is a small sample in single center. In addition, G401 (rhabdoid tumor of the kidney cell line) was formerly classified as WT cell lines, but they have had more correct classification since [31]. Hence, multicenter, large sample, and accurate cell model are the direction of further research.

## 5. Conclusion

Our study showed that there were significant differences in the expression levels of TGF-*β*1 and P-smad2/3 between WT and adjacent normal tissues. The activation of TGF-*β*/Smad was strongly linked with WT clinical stage and metastasis. Moreover, T*β*RI inhibition significantly decreased WT migration, invasion, and growth in vitro and repressed TGF-*β*/Smad signaling-mediated EMT. Overall, these findings may increase the understanding of WT tumorigenesis and improve diagnosis and targeted individualized treatment.

## Figures and Tables

**Table 1:** Correlation analysis between TGF-*β*1 and P-smad2/3 expression and the clinicopathological characteristics of Wilms' tumor in 60 patients.

Characteristics	Total number (*n* = 60)	TGF-*β*1		*X* ^2^	*P* value	P-smad2/3		*X* ^2^	*P* value
		Positive	Negative			Positive	Negative		
Age									
0-3	39	24	15	0.155	0.694	17	22	0.003	0.956
>3	21	14	7			9	12		
Gender									
Boys	35	21	14	0.402	0.526	15	20	0.008	0.930
Girls	25	17	8			11	14		
Pathology									
FH	47	30	17	0.023	0.879	19	28	0.747	0.387
uFH	13	8	5			7	6		
Clinic stage									
I-II	22	10	12	4.781	0.012	5	17	6.007	0.014
III-IV	38	28	10			21	17		
Metastasis or invasion									
Yes	24	19	5	4.381	0.038	16	8	8.869	0.003
No	36	19	17			10	26		

## Data Availability

The data of this study are available from the corresponding author upon request.
